# Self-Reported Tianeptine Experiences on Reddit: Natural Language Processing–Assisted Qualitative Study

**DOI:** 10.2196/86683

**Published:** 2026-07-07

**Authors:** Christopher J Counts, Anthony V Spadaro, Sahithi Lakamana, Abeed Sarker, Rachel Wightman, Jennifer Love, Diane Calello, Jeanmarie Perrone

**Affiliations:** 1 Department of Emergency Medicine, Rutgers New Jersey Medical School, 140 Bergen St, Newark, NJ, 07103, United States, 1 720-446-8235; 2 Department of Biomedical Informatics, School of Medicine, Emory University, Atlanta, GA, United States; 3 Department of Emergency Medicine, Brown University Health, Providence, RI, United States; 4 Department of Emergency Medicine, Icahn School of Medicine at Mount Sinai, New York, NY, United States; 5 Department of Emergency Medicine, Perelman School of Medicine, University of Pennsylvania, Philadelphia, PA, United States

**Keywords:** tianeptine, Reddit, social media, opioid use disorder, natural language processing, surveillance

## Abstract

This study, using natural language processing and manual thematic analysis of Reddit posts, revealed a rapid rise in discussions about tianeptine, with posts frequently reporting dependence, withdrawal, and coingestion with other unregulated substances, highlighting tianeptine as an emerging public health concern.

## Introduction

Tianeptine is an atypical antidepressant that modulates glutamate activity and, at high doses, agonizes mu opioid receptors. Although not approved by the US Food and Drug Administration, it is sold in unregulated marketplaces such as gas stations, convenience stores, smoke shops, and online. Reports suggest that individuals may use tianeptine recreationally or to manage pain, opioid use disorder (OUD), depression, or anxiety [1,2]. In countries where tianeptine is an approved and regulated antidepressant, its use has been linked to dependence and harm, leading several countries to classify it as a controlled substance. The population using tianeptine outside of formal medical settings remains poorly characterized. Social media platforms such as Reddit offer large, publicly available corpora of anonymous narratives that can support toxicovigilance when combined with natural language processing (NLP) methods [3,4].

This study used NLP approaches for data processing and manual coding for thematic analysis to characterize tianeptine-related discourse. The research questions were as follows: (1) How have posts mentioning tianeptine and related keywords changed over time? and (2) What themes characterize self-reported experiences with tianeptine?

## Methods

### Data Source and Identification of Tianeptine-Related Posts

We collected all Reddit data from 2005‐2024 via Pushshift (2005‐2023) and the Python Reddit API Wrapper (2024) We identified approximately 9000 drug-related subreddits to ensure discussion relevancy and reduce potential noise introduced by ambiguous/polysemous expressions. We searched for the keyword *tianeptine* in the collected data to identify relevant posts. We used a word2vec model [5] to identify expressions, including multiword expressions, that are semantically similar to *tianeptine*, and manually selected relevant ones. The final keyword set included *tianeptine*, *Neptune’s Fix*, *Neptune’s Elixir*, *Zaza*, *Zaza Red*, *Pegasus*, *Pegasus Red*, *Tianaa*, *TD Red*, *gas station heroin*, and *gas station dope.* Neptune’s Fix Elixir, Zaza, Tianaa, Pegasus, and TD Red are common brands of tianeptine products. For potentially ambiguous terms (eg, “Pegasus”), we manually reviewed random samples to confirm relevance to tianeptine and excluded terms generating frequent false positives. Posts were grouped by year and keyword to describe trends over time. Counts for 2024 are reported descriptively but excluded from the trend figure.

### Manually Coded Thematic Analysis

From 19,188 tianeptine-mentioning posts across 9707 subreddits, we drew a random sample of 200 posts for qualitative analysis. This analysis is described in detail in Multimedia Appendix 1. Briefly, 3 authors with medical toxicology expertise conducted a preliminary open-coding exercise and noted recurrent topics, experiences, and patterns related to tianeptine use. The group then met to compare preliminary codes and organize them into broader conceptual categories. Through repeated discussion, the authors created a structured codebook specifying, for each theme, a brief descriptive label, an operational definition, criteria for inclusion, and one or more example summaries drawn from representative posts. The codebook was iteratively revised across multiple coding cycles until no additional themes were identified (see Multimedia Appendix 1 for the full codebook). After the final codebook was established, 2 reviewers independently coded all posts, with disagreements resolved through discussion and, when needed, adjudication by a third team member.

To assess interrater reliability, 2 coders independently applied the final 8-theme codebook to a random subset of 50 posts (25% of the qualitative sample). For each theme, we treated coding as a binary present/absent decision and calculated the Cohen κ based on a 2×2 contingency table of coder decisions.

### Ethical Considerations

This study analyzed secondary data from only publicly available Reddit posts and did not involve interaction with individuals. Usernames and URLs were not included in the analysis. All examples are paraphrased to reduce reidentification risk. Additional methodological detail is provided in Multimedia Appendix 1.

## Results

### Frequency and Trends

**Figure 1.**
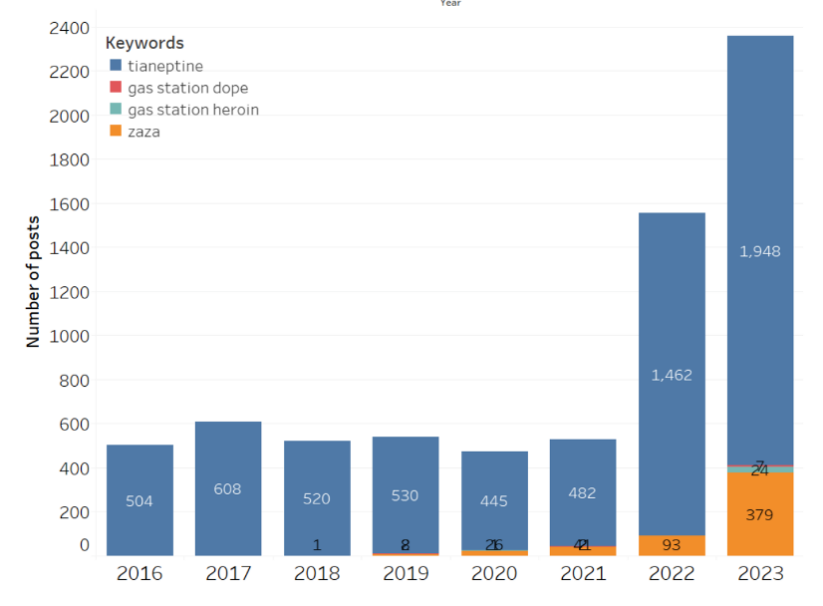
Tianeptine and select keyword frequency trend (2016‐2023).

We identified 19,188 posts from 9707 subreddits in 2005‐2024. Approximately 26% (4912/19,188) were posted in the last 4 years. Annual counts increased sharply from 1555 (2022) to 2358 (2023; Figure 1). Posts referencing *Zaza* rose from 26 (2020) to 379 (2023). Comentioned substances included kratom (n=328), buprenorphine (Suboxone) (n=139), gabapentin (n=121), and pregabalin (n=75).

### Manually Coded Themes

Among 200 sampled posts, 8 themes were identified (Table 1).

**Table 1.** Manually coded thematic analysis.

Theme	Theme frequency (n=200), n (%)	Paraphrased example
Withdrawal symptoms and management	68 (34)	A poster described experiencing cold sweats, diarrhea, confusion, panic, nausea, and headache within 24 hours of stopping tianeptine after a period of regular use. They attempted to manage symptoms with kratom.
Tianeptine dependence	56 (28)	A poster described an “insane rush” followed by the disappearance of all their problems and worries after their first time using tianeptine. After experiencing some hardships, they began using tianeptine daily and spending a significant amount of money on it.
Tianeptine use and opioid use disorder	16 (8)	A poster with a history of opioid use disorder reported discontinuing buprenorphine maintenance therapy and substituting tianeptine products. After experiencing withdrawal, they asked whether it would be safe to restart buprenorphine immediately.
Substances coingested	12 (6)	A poster described potentiating the effects of tianeptine by taking it with pregabalin, clonazepam, lorazepam, and dextromethorphan.
State-level bans	12 (6)	After a state implemented a tianeptine ban, a poster described visiting multiple retailers that had previously sold products “under the counter,” only to find all supplies depleted. They discussed ordering online from out-of-state vendors and considered traveling to a neighboring state.
Use for pain and depression	9 (4.5)	A poster with chronic gastrointestinal symptoms described using tianeptine when prescription medications and standard antidepressants provided insufficient relief, framing it as a way to “take the edge off” and improve daily functioning.
Adverse effects	7 (3.5)	A poster who had taken tianeptine regularly for one year inquired about tingling in the face and throat, nausea, and bodily sensations of heat and cold.
Suspected adulteration	1 (0.5)	A poster who had taken the same tianeptine brand for months reported suddenly experiencing severe effects from a new batch that resulted in hospitalization, raising concern that the product might be adulterated with a synthetic cannabinoid or other substance.

#### Withdrawal Symptoms and Management

Withdrawal symptoms and management strategies were most frequently mentioned (n=68, 34%). Posters described gastrointestinal symptoms, sweating, dysphoria, restlessness, and sleep disruption, comparing their experiences to opioid withdrawal (n=34, 17%). Many described self-directed tapering or switching to other substances (n=23, 11.5%).

#### Dependence

Tianeptine dependence was coded in 56 posts (28%). Posters recounted escalating doses, difficulty controlling use despite adverse consequences, and financial strain (n=44, 22%). Some described hospitalizations after ingesting gram-level doses of tianeptine (n=3, 1.5%).

#### OUD

Sixteen posts (8%) discussed tianeptine use in the context of OUD, including transitioning from prescription opioids or heroin to tianeptine, or using buprenorphine to manage tianeptine withdrawal. Kratom was the most frequently comentioned substance (n=38, 19%).

#### State-Level Bans, Adverse Effects, and Suspected Adulteration

Twelve posts (6%) addressed state-level bans, including abruptly losing access and turning to online vendors. Some described concern about phenibut withdrawal from coformulated products (n=6, 3%). Seven posts (3.5%) focused on adverse effects, and one described suspected adulteration.

#### Reliability Analysis

Interrater reliability for thematic classification was moderate overall. The mean Cohen κ across themes that occurred at least once in the reliability subset was 0.52, with a mean raw agreement of 89%. For withdrawal symptoms and management, agreement was 90% (κ=0.72); for dependence, 82% (κ=0.48); for OUD, 84% (κ=0.29); for coingestants, 80% (κ=0.52); for state-level bans, 100% (κ=1.00); for use for pain and depression, 92% (κ=0.62); and for suspected adulteration, 100% (κ=1.00). No posts in this subset were coded with the theme of adverse effects by either coder, so interrater reliability could not be meaningfully estimated for that theme.

## Discussion

Reddit discussion of tianeptine increased markedly in 2022‐2023. Thematic analysis highlighted dependence, withdrawal management, and discussions of other substances. Reports of buprenorphine use to manage withdrawal align with case reports, though posters frequently described unproven strategies, underscoring the need for improved access to evidence-based addiction treatment.

Frequent comentions of kratom and gabapentinoids suggest overlapping populations engaged in polysubstance use. Posts describing abrupt withdrawal after state scheduling illustrate possible unintended consequences of rapidly changing drug markets, though these data are descriptive and cannot establish causality.

Several posts provide insight into the impact of state-level scheduling of tianeptine as a controlled substance. Individuals may acquire tianeptine from alternative markets or may experience abrupt withdrawal. One post from October 2023 described an experience with the tianeptine product Neptune’s Fix in which the poster suspected adulteration of the product, describing an “alternative cannabinoid high.” Samples of this product were later analytically confirmed to be adulterated with synthetic cannabinoids. This finding was reported in the New York Times on January 10, 2024, and in the literature on February 1, 2024 [6,7].

This study suggests that applying NLP for data processing alongside manually coded thematic analysis of Reddit posts may be useful for toxicosurveillance. Recent work using transformer models and large language models has shown that automated pipelines can classify and track opioid-related social media chatter at scale, extending earlier machine learning–based toxicovigilance approaches. Additionally, social media data can capture clinically relevant signals about mental health, reinforcing the value of online platforms for population-level surveillance of risk and vulnerability [8-10]. Our findings add to this literature by focusing on self-reported experiences with an emerging drug.

Limitations include Reddit’s nonrepresentative demographics, self-reported data lacking clinical verification, coding of only 200 randomly selected posts, and the descriptive design precluding causal inference. Additionally, changes to the data available for 2024 limited analysis to posts through 2023, which may limit temporal generalizability. Nonetheless, the observed increase in tianeptine-related posts, coupled with frequent self-reported dependence and withdrawal, reinforces tianeptine as a drug of public health concern. Simple NLP-assisted approaches may help monitor evolving substance use patterns in near real time.

## Supplementary material

10.2196/86683 Multimedia Appendix 1 Methods for data collection and natural language processing and details of qualitative thematic analysis.

## Abbreviations:

NLP: natural language processing
OUD: opioid use disorder
