# Supplementary material for: Self-Reported Tianeptine Experiences on Reddit: Natural Language Processing–Assisted Qualitative Study
Source: JMIR Infodemiology. 2026 Jul 7;6:e86683. doi: 10.2196/86683 (PMC13340079; doi:10.2196/86683)
Supplement: Multimedia Appendix 1 [file infodemiology-v6-e86683-s001.docx]

**e-Supplement**

**Expanded Methods**

**1. Data Collection and Natural Language Processing**

**1.1 Reddit data sources**

We analyzed publicly available Reddit posts mentioning tianeptine or related terms from January 1, 2005, to December 31, 2024. Data from 2005 to 2023 were obtained from the Pushshift application programming interface (API), which archived historical Reddit submissions. Data from 2024 were collected using Reddit’s official API via a Python wrapper because Pushshift access became restricted.

For 2005–2023, we accessed all publicly available posts; in 2024, data collection was rate-limited and therefore restricted to a pre-specified set of drug-related subreddits only (n = 9000), and not all available data could be collected. Metadata fields included post ID, subreddit name, timestamp, title, and text body.

**1.2 NLP methods and models**

NLP methods were employed to semi-automatically identify name variants of tianeptine. Specifically, a word2vec model trained on drug-related social media data was used to capture semantic representations of phrase-level expressions. The strategy for creating a large corpus of drug-related chatter is provided in a prior publication [1]. The model was trained using a context window size of 9 and each vector in the model was represented using 400 dimensions. These parameters were chosen based on findings of prior work that demonstrated effectiveness for the task of drug misspelling generation from social media data [2]. Prior to model training, texts were preprocessed by lowercasing and removing punctuations. No lemmatization or stopword removal was performed. Multi-word expressions (e.g., gas station heroin) were identified using the method proposed by Mikolov et al. (2023), which identifies phrases by comparing the co-occurrence frequency of two words against their individual frequencies, adjusted by a discounting factor to prevent forming phrases from very rare words. Expressions occurring less than 10 times in the corpus were discarded.

Following the training of the model, it was queried iteratively to identify possible name variants and misspellings of tianeptine. In the initial iteration, the term ‘*tianeptine*’ was used, and a list of the most semantically similar expressions was generated by the model based on cosine similarity. The top 50 expressions were manually reviewed to identify true variants and misspellings. Since word2vec captures semantic similarity based on context, the most similar expressions typically included drugs similar to tianeptine (*e.g.*, other psychoactive substances such as *kratom*), along with name variants for tianeptine (*e.g.*, *zaza*) and its common misspellings (*e.g.*, *tienaptine*). In subsequent iterations, identified variants were used for querying the model in an identical fashion. The process was repeated until no new variant was found.

The flowchart in Figure S1 illustrates the steps involved in this study and the number of Reddit posts involved in each. Expression variants for tianeptine were used to search through the retrieved posts using regular expressions in Python. Small samples of posts matched by the expressions were manually reviewed to ensure high signal-to-noise ratio, particularly for ambiguous/polysemous expressions. Since the subset of subreddits included in the study was drug-related ones, the identified expressions typically represented tianeptine.


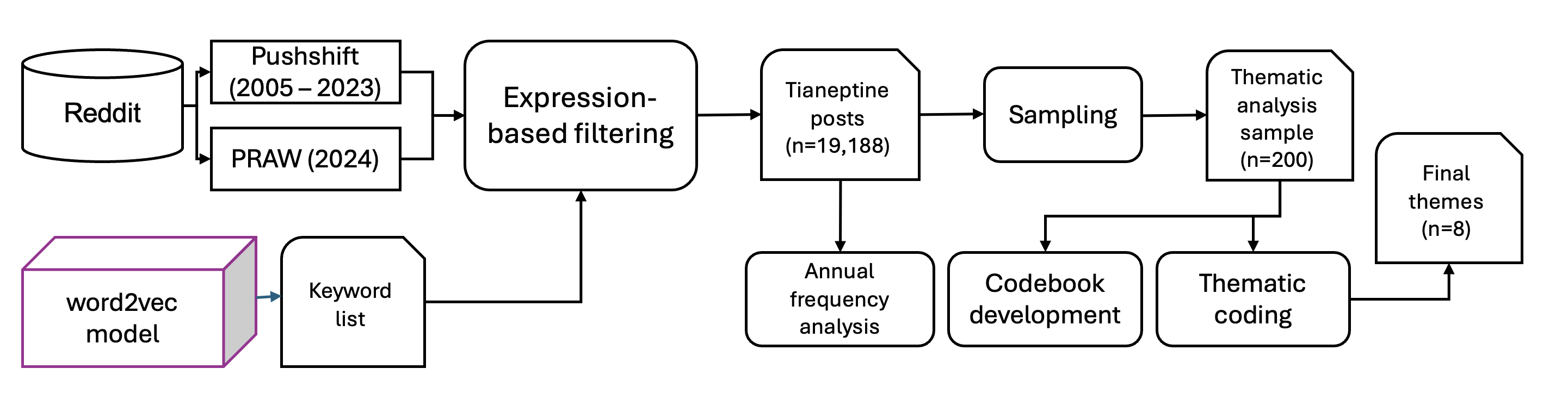


**Figure S1.** Study flowchart –from data collection to thematic analysis of a sample. Relevant numbers are presented for each step.

**2. Qualitative Thematic Analysis**

**2.1 Sampling strategy**

From the full set of 19,188 tianeptine‑related posts, we drew a simple random sample of 200 posts for qualitative analysis. Sampling was performed after deduplication of posts and restriction to English‑language content. Posts that consisted solely of URLs, images, or non‑text content were excluded from the sampling frame.

The target sample size (n=200) was chosen a priori as a balance between diversity of content and feasibility for in‑depth manual coding. During coding, the research team monitored for the emergence of new themes and determined that thematic saturation had been reached within this sample.

**2.2 Codebook development**

Three authors with training in medical toxicology conducted a preliminary open‑coding exercise on an initial subset of 50 randomly selected posts. Each coder independently reviewed the same set of posts and noted recurrent topics, experiences, and patterns related to tianeptine use.

The team met to review these initial codes and to group them into higher‑order categories. Through iterative discussion, the authors developed a structured codebook that included:

- A short label for each theme
- An operational definition
- Inclusion elements
- One or more example summaries drawn from posts

This codebook was refined over multiple rounds of coding until no new themes emerged.

**2.3 Coding procedure and agreement**

Using the finalized codebook, each of the 200 posts was coded independently by two members of the team. Posts could be assigned multiple themes where appropriate (eg, dependence and withdrawal management in the same narrative).

Coding was conducted in a tabular format that included the post ID. After independent coding, discrepancies were identified and discussed in consensus meetings. Disagreements were resolved through discussion; when necessary, a third coder adjudicated.

**2.4 Paraphrasing for reporting**

To reduce the risk of re‑identification, we did not include verbatim text from Reddit in the manuscript or this appendix. Instead, the examples below are paraphrased and may merge details from multiple posts while preserving the underlying meaning and context.

**2.5. Qualitative Codebook**

**1. Withdrawal symptoms and management**

- **Definition:** Posts describing physical or psychological symptoms experienced when reducing or stopping tianeptine, and/or strategies used to manage these symptoms.
- **Key elements:** Explicit mention of stopping, tapering, running out of product, or “withdrawal”; descriptions of symptom clusters (eg, gastrointestinal upset, chills/sweats, insomnia, restlessness, mood disturbance); mention of self‑treatment approaches.
- **Paraphrased example:** A poster reports that within a day of stopping high‑dose capsules, they developed intense stomach cramping, diarrhea, sweats, anxiety, and an inability to sleep, and describes trying over‑the‑counter medications and other substances to “take the edge off.”

**2. Tianeptine dependence**

- **Definition:** Posts describing loss of control over tianeptine use, escalating doses, persistent craving, or continued use despite negative consequences.
- **Key elements:** Narratives of dose escalation, difficulty cutting down, feeling “hooked” or “addicted,” financial or social harms attributed to ongoing use.
- **Paraphrased example:** A poster explains that what began as a few pills a day progressively escalated to large daily quantities, with most of their disposable income going toward tianeptine, leading to debt and conflict with family but ongoing use despite these harms.

**3. Tianeptine use in the context of opioid use disorder**

- **Definition:** Posts in which tianeptine use is discussed specifically in relation to a history of opioid use disorder (OUD), opioid treatment, or transition from other opioids.
- **Key elements:** Explicit mention of OUD, methadone, buprenorphine/Suboxone, heroin, or prescription opioids; tianeptine framed as a replacement, bridge, or relapse trigger.
- **Paraphrased example:** A poster with prior heroin use describes stopping buprenorphine, switching to tianeptine capsules purchased at a gas station, and later asking whether it is safe to restart buprenorphine while experiencing tianeptine withdrawal symptoms.

**4. Substances co‑ingested**

- **Definition:** Posts that focus on tianeptine taken together with other substances (eg, benzodiazepines, gabapentinoids, dextromethorphan, alcohol) or used alongside multiple agents to manage withdrawal or enhance effects.
- **Key elements:** Detailed “recipes” or combinations; descriptions of synergistic or unexpected effects; emphasis on stacking substances.
- **Paraphrased example:** A poster describes taking tianeptine with dextromethorphan and diphenhydramine to intensify euphoria.

**5. State‑level bans and market changes**

- **Definition:** Posts discussing state regulations or local policy changes affecting tianeptine availability, and users’ responses to these changes.
- **Key elements:** References to specific states, new scheduling decisions, products being removed from stores, or advice on where to obtain remaining stock.
- **Paraphrased example:** A poster from a state that recently scheduled tianeptine recounts going from shop to shop only to find that most had stopped selling their usual brand, until finding a shop that had a supply “under the counter” available for sale.

**6. Use for pain, mood, or other indications**

- **Definition:** Posts that describe tianeptine used primarily for pain relief, mood or anxiety symptoms, or functional improvement rather than explicitly for intoxication.
- **Key elements:** Tianeptine framed as “medication,” “antidepressant,” or a way to “take the edge off” chronic symptoms; sometimes contrasted with prescribed treatments that the poster feels are insufficient.
- **Paraphrased example:** A poster with chronic anxiety and depression uses tianeptine to self-medicate these symptoms.

**7. Adverse effects**

- **Definition:** Posts focusing on acute or chronic adverse effects attributed to tianeptine.
- **Key elements:** Descriptions of symptoms arising during or after tianeptine use (eg, paresthesias, cardiovascular complaints, neurologic changes), and references to emergency department visits.
- **Paraphrased example:** A poster who has used tianeptine for months describes the development of an unpleasant “comedown” that lasts four to five hours after every dose.

**8. Suspected adulteration**

- **Definition:** Posts focusing on concerns that a tianeptine product may be contaminated, adulterated, or otherwise different from expected (eg, unusual potency, color, taste, or effects).
- **Key elements:** Expressions of concern about product quality or additional ingredients, reports that a familiar product suddenly “feels different,” and comments linking these changes to new or unexpected symptoms.
- **Paraphrased example:** A poster reports that a recent batch of their usual tianeptine product has a different appearance and caused severe atypical effects, leading them to suspect it contains other substances.

**References**

[1] Sarker A, Gonzalez G. A corpus for mining drug-related knowledge from Twitter chatter: Language models and their utilities. Data Brief. 2016 Nov 23;10:122-131. doi: 10.1016/j.dib.2016.11.056. PMID: 27981203; PMCID: PMC5144647.

[2] Sarker A, Gonzalez-Hernandez G. An unsupervised and customizable misspelling generator for mining noisy health-related text sources. J Biomed Inform. 2018 Dec;88:98-107. doi: 10.1016/j.jbi.2018.11.007. Epub 2018 Nov 13. PMID: 30445220; PMCID: PMC6322919.

[3] Mikolov T, Sutskever I, Chen K, Corrado GS, Dean J. Distributed representations of words and phrases and their compositionality. Adv Neural Inf Process Syst. 2013;26:3111-3119.
